# Supplementary material for: Law and (rec)order: Updating memory for criminal events with body-worn cameras
Source: PLoS One. 2020 Dec 16;15(12):e0243226. doi: 10.1371/journal.pone.0243226 (PMC7743977; doi:10.1371/journal.pone.0243226)
Supplement: Attachment — Submitted filename: BWC response to review letter.docx [file pone.0243226.s001.docx]

**Reviewer 1:**

***(1) My main concern here is about statistical power, which given the sample size is relatively low for detecting modest effects. I'd suggest that at the least, the authors give this issue some direct treatment in the paper, consider the kinds of effect sizes their study could reliably detect, and moderate some of the claims accordingly.***

A power analysis using G*Power3.1 revealed that 84 participants would be needed, given an effect size of .4, with a level of significance at .05 (Faul, Erdfelder, Buchner, & Lang, 2009). However, more participants would be required to detect smaller effects. We have added a statement in the limitations section of the discussion that addresses the statistical power. On page 27 we have added, “The current study is limited by the sample. Having a relatively modest sample size limited the power of our analyses, particularly in relation to the critical interaction between the review and statement timing condition.”

***(2) There are a few places where the wording could be somewhat clearer. In particular (a) the opening few sentences of the introduction; (b) the wording of Hypothesis 3 on p.10, and (c) the paragraph beginning "To assess..." that starts at the bottom of p.17.***

To provide clarity for readers, points (a) to (c) have in turn been addressed.

A) We have clarified the wording of the first two sentences. We thank the reviewer for this suggestion and we think the introduction is much clearer now.

The manuscript now reads, “It is commonly believed that we encode and retrieve details in memory similar to the way a camera records and replays video footage [1, 2]. Consequently, eyewitness testimony is one of the most persuasive forms of evidence for juries [3].

B) More detail has been included on page 10 to clarify the third hypothesis. The hypothesis now reads, “We predicted that participants who reviewed their footage would be less likely to include off-camera details, except for those who provided an initial statement before review (Hypothesis 3). That is, we believed the opportunity to prepare an initial statement may preserve any “off-camera” details participants could recall before they were shown what they had recorded on camera for the first time.”

C) The wording of this paragraph has been simplified and cut. It now reads, “We further performed a 2 (statement timing) x 2 (review) mixed-design ANOVA on the proportion of off-camera details within initial and final statements.” We hope that readers understand that the purpose of this analysis was to examine how inclusion of ‘off-camera’ details had *changed* from initial to final statement. The inclusion of Figure 2 within the manuscript should further help to illustrate these findings.

***(3) The authors might be interested to look at and cite a recent paper by Henkel & Milliken, which seems relevant to their aims and hypotheses:*** [***https://doi.org/10.1016/j.jarmac.2020.07.002***](https://doi.org/10.1016/j.jarmac.2020.07.002)

Thank you for this recommendation. We have now cited Henkel and Milliken (2020), which is very relevant to the current study given the similarities between the investigation of ‘intact’ and ‘removed’ details on ‘cropped’ photographs and our study’s investigation of on- and off-camera details.

On page 8 we have added the following, “A recent study required individuals to edit a photograph by cropping its parameters then review this edited version several days later. The findings revealed participants simultaneously experienced enhanced recall for intact details yet impaired recall for removed details during a final recall test one week after taking the original photograph.”

***(4) I didn't seem to be able to find Figure 1***

Please accept our apologies that Figures 1 and 2 were omitted from the submission of this manuscript. Both of these are now included and should provide greater clarity for readers as to the design of the study (Figure 1) and our findings regarding ‘off-camera’ details included within initial and final statements (Figure 2).

***(5) I was interested in the authors' justification for their control tasks - having people describe the 'mountain' video instead of the crime event, and also having some people review a nature documentary. I wonder whether the inclusion of these control tasks was necessary, and whether they might even have affected the results. In many studies like this, a control task is necessary to avoid the possibility that, for example, the experimental task acts as a distractor, or takes longer. But in this study there seemed no need to control for this possibility. I worry that the control tasks could have artificially weakened performance in the control conditions by preventing rehearsal of the film, for example.***

We acknowledge the possibility that this study could indeed run without any control tasks, however, the two tasks allowed us to control for confounding variables of time or the activity completed to ensure all participants had a similar experience regardless of their assigned condition.

The first control task required participants to describe a landscape that all participants saw in virtual reality during the first session. This task was given to participants in the ‘no initial statement’ condition whilst those in the ‘initial statement’ recalled the investigation they undertook. Instructions for this control task informed participants they would be discussing the scene they viewed *before* they participated in the police investigation. This was an animated landscape without any people present, which clearly differed from the real-life setting of the investigation. This task ensured participants in the no initial statement condition were also recalling an event from one week ago.

The second control task was included for those in the ‘no review’ condition. Whilst reviewers watched their chest-camera footage, we ensured our control condition also involved watching a video to eliminate the possibility that the mere act of taking time to watch a video somehow influenced recall. Similar to the ‘mountain landscape’ control task, we wanted to include an activity of a similar length for non-reviewers but that was entirely unrelated from the contents of the investigation. This was done to prevent any kind of confusion for participants about which video they were asked to describe in their statements. Furthermore, in the control video there was no content that may have prompted memory for details of the investigation in some way.

Participants had an unlimited amount of time to complete their free recall. Therefore, we hope that this allowed all participants to spend as long as they wished on rehearsing or remembering details of the incident.

***(6) The authors need to include the descriptive data in Tables 2-4 (Table 1 doesn't seem to exist) separated out by initial statement condition (i.e. for each cell in their 2x2 design), not just collapsed across these conditions - regardless of whether the effects were significant.***

The labelling of tables has been amended and now presents Tables 1 – 3, and any in-text references to these tables have been adjusted accordingly. Full descriptive data has now been provided in Tables 1 – 2 (pages 16 and 17) to include initial statement conditions in line with the suggestion. This issue was not applicable to Table 3 which only includes the data of those in the initial statement condition.

***Reviewer #2:***

***I am concerned about the applied nature of the literature and tone of the paper: positive but actually rather contentious in terms of not accentuating the importance of confidence and how video footage can undermine one’s confidence, resulting in altering a perfectly good statement of ‘what I remember and what I noticed’ because of what the ‘video says’ and/or ‘doesn’t say’ and the impact of retrieval environment/method.***

Thank you for highlighting the importance of participant confidence and whether reviewing body-worn video may in fact reduce confidence in how they remembered the event due to differences between camera and memory.

To provide further information, after completing both initial and final statements, participants rated their agreement with the following statements:

*1. I was motivated to provide the most complete and accurate statement possible.*

*2. I am confident that the details in my statement are correct.*

*3. My statement includes all the details I can remember from the event.*

*4. I feel I could mentally place myself at the event I described*

*5. My statement includes some details that I do not explicitly remember but assumed must have happened.*

*6. The video I watched today was helpful for writing my statement.*

Participants in the review condition further rated their agreement with the following:

*7. The video was a true reflection of what I saw last week.*

Scale: (1) Strongly Disagree - (5) Strongly Agree

One reason for including these statements was for us to learn about participant confidence and begin to unpick the ‘what I remember’ versus ‘what I noticed because of what the video says’ element you highlight. These statements can be found via <https://osf.io/xbtvf/>. We believe statements 2, 5 and 7 best address your concerns so each will be discussed in turn.

Statement 2 (the results of which are included on page 20) allowed us to see that reviewers were significantly more confident in their accuracy than non-reviewers.

In response to this suggestion, we performed a 2 (review) x 2 (statement timing) mixed-design ANOVA to understand the difference between reviewer’s and non-reviewer's confidence in the accuracy of their initial *and* final statements. The findings revealed no significant interaction effect, and therefore it cannot be concluded that reviewer’s confidence was significantly higher after their final statement (once they had reviewed footage) compared to their initial statement. In the manuscript, we highlight our ANOVA findings contrasting all four conditions with regards to their confidence ratings after they completed their final statement. Here, we reported a significant main effect of the review condition on confidence. Together, these findings seem to suggest reviewers did not show increased confidence following a process of review and amending their statement. Therefore, this significant main effect is likely driven by the confidence of reviewers who never produced an initial statement compared to non-reviewers. We have since included the findings of the mixed-design ANOVA into our results section (page 20-1).

We have also interpreted our findings on participant confidence more within the discussion (Page 24). In relation to your concern, our confidence scale does not explicitly tap into whether participants retrospectively lost confidence in what they originally wrote in their initial statement before reviewing their footage. Moreover, we cannot understand reasons behind these confidence ratings (i.e. ‘because the camera doesn’t lie’, original memory has been boosted, there are details the participant didn’t remember before, or consistency between the two sources).

The purpose of statement 5 was to measure whether reviewers would include details in their statement because ‘the video says so’. For brevity, we did not include data for all statements within the manuscript, but in response to your suggestion, we did not find a significant difference between reviewers and non-reviewers with regards to agreement with this statement.

Finally, statement 7 allowed us to measure participants’ potential awareness of discrepancies between their chest-footage and what they saw themselves during the investigation. The mean agreement rating was 3.56 (*SD* = 1.50) indicating that participants were, on average, mostly in agreement that their video reflected their true experience. This finding is similar to Jones, Crozier, and Strange’s (2018) where participants believed body-worn video was an extension of an officer’s experience.

***A misinformation condition would really improve this work, in my opinion. Readers will find this interesting, and the work is of broad interest.***

Whilst including a misinformation condition would be theoretically interesting, we decided not to include one in the current study due to the applied nature of the research. We wanted to be able to generalise the findings to policing practice as one of the first research studies into body-worn cameras and memory. Presumably, it would be rare for police officers to view body-worn video that contained purposefully false information (i.e. footage that had been tampered with or onscreen details that were not present in the original scene).

However, to a certain extent we *did* investigate memory for misinformation. In our study, participants viewed BWC footage that varied in the extent to which it differed from the incident they would have seen (i.e., if their head was faced in a different direction to their chest). While this footage was true to the event, it can be considered misinformation because it is not true to what the participant viewed at the time.

Another interpretation of 'misinformation’ may be analogous to instances where an officer reviews a colleague’s footage, similarly showing a different perspective to the one they experienced and thus details they did not see which may be incorporated into their own memory for events. We believe this provides grounds for an interesting future direction.

***Concerns***

***i) The paper is firmly situated in in the context of police and investigation, yet participants are all UG students who clearly differ from police professionals.***

Potential disparities between policing professionals and our undergraduate student sample have been addressed within the “limitations and future directions” section (see Page 27). Within the same paragraph, we have also included the following limitation which ties in with your suggestion about police officers not wanting to underperform or deviate from their camera footage: “It is possible that officers may feel more inclined to produce reports that are identical to their footage due to fears of scrutiny (something we perhaps would expect less of our student participants) [98].”

***ii) The authors have used a written format questionnaire type document to collect the event information, which included some cues, but this deviates from applied practice (in the UK, that is).***

In this study we used a guided recall strategy that has been used in a number of studies (e.g. Harkness, Paterson, Denson, Kemp, Mullan & Sainsbury, 2014; Paterson, Kemp & McIntyre, 2012; Paterson, Eijkemans & Kemp, 2015). Specifically, they were instructed, “*“Please report as much information as you can remember about this investigation below (e.g. who was present, the setting, objects, actions and dialogue), even if a detail seems trivial.”* While police officers would not normally have additional cues (e.g., person, setting) they would know what was expected from them in a report. We gave these additional cues to our undergraduate participants, so they had a better idea about what to report. In the UK, witnesses can be given a self-administered interview (SAI©; Gabbert, Hope, and Fisher, 2009) to complete. The SAI© is a guided recall tool that elicits written responses about the event, perpetrator, scene, witnesses, and vehicles that were present.

***iii) I am unsure as to the rationale for having a 1-week delay between encoding and retrieval. I may have missed the rationale for this, but this is at odds with police practice, generally. Please clarify.***

Whilst ideal policing practice would involve reports being completed immediately following attendance of an incident, the one-week delay included in the current study considers that this may not always be possible given a police officer’s workload and prioritisation of tasks. Police Officers in the United States noted that the high volume of calls received often meant that organising and tagging recorded video was not completed immediately following an incident (Gaub, Todak, & White, 2018), let alone reviewing. Furthermore, in some jurisdictions, police forces require their officers to view footage before they complete their written statement. For example, New South Wales Police Force in Australia outline the following in their Standard Operating Procedure (2018, p. 14):

*“Police should view BWV footage of an incident and interactions with witnesses, prior to preparing police or witness statements.”*

Regarding critical incidents, the guidelines read:

*“Police directly involved in a Critical Incident should be provided the opportunity to view relevant BWV footage prior to being interviewed.”*

It has also been reported that the Crown Prosecution Service in the U.K. often does not request a statement from an officer until months later (Rowe, Pearson, & Turner, 2017). In these cases, body-worn video can become crucial for cuing memory and an officer can replay footage repeatedly in preparation for being interviewed about an event.

***iv) The finding ‘analyses revealed a significant effect of review condition on the completeness of statements, F(1,93) = 22.72, p377<.001, partial η2= .196, where those who reviewed their body-worn video included more details (both correct and incorrect)’ was in my view to be expected, likewise the accuracy and detail results. The big questions that arise here are, is this improved memory performance, or is this simply a case of altering accounts because video’s don’t lie, so I must be wrong, and/or a loss of confidence in my own memory and not wanting to appear to underperform (which is laid bare given the fact that video footage is available). This research raises this very significant question, but does not in my opinion answer this question.***

This is a very important question, however it is beyond the scope of the current research. We have taken the first step of establishing that camera conformity exists, which has very important practical implications. The next step is to investigate the mechanisms responsible for this (i.e., whether it is due to changes in memory or simply changes in reporting). Future research may consider employing source monitoring tasks or instructions to report only on the basis of what they remember from the VR experience (and not from the recording).

In our study, participants were instructed to report what they could recall from the investigation itself, therefore they were made aware it was their memory we were interested in. Prior to completion of the free recall statement, participants received the following instructions onscreen:

*“One week ago, you completed an investigation of a venue as part of your duties as a police officer. This involved you watching a video in virtual reality.*

*Please report as much information as you can remember about this investigation, even if a detail seems trivial. Your account should be as complete and accurate as possible, but you are instructed to avoid guessing.”*

The instructions on the free recall template itself further read:

*“Please report as much information as you can remember about this investigation below (e.g. who was present, the setting, objects, actions and dialogue), even if a detail seems trivial. Your account should be as complete and accurate as possible, but you are instructed to avoid guessing.”*

***v) The finding that ‘participants who did not review their body-worn video included a higher proportion of off-camera details in their final statements, M=0.08 (0.10) than participants who reviewed their body-worn video, M= 0.04 (0.06)’ speaks to the above question regarding confidence and altering in line with a ‘video does not lie’ hypothesis. Likewise, ‘a larger proportion of ‘off-camera’ details in their initial statement than in their final statement’. I note that a confidence scale was used, which supports my concerns.***

Thank you for highlighting the important and relevant issue of whether individuals trust their camera footage more than their episodic memory, as opposed to the camera footage in fact changing their memory for the event. For instance, we acknowledge this issue within the manuscript on page 24: “Alternatively, it is plausible that rather than body-worn video changing episodic memory, participants merely altered their written statements to correspond with their footage. Therefore, the alternative perspective offered by an individual’s footage may shape what they choose to recall. We believe this aspect is captured in both of your concerns (iv) and (v), and within your final point under ‘additional concerns’ therefore all will be addressed here.

On page 20 of our results section, we indicated that confidence in details included within statements was higher for reviewers than non-reviewers. This perhaps leads readers to a question of whether greater confidence arises as a result of the number of off-camera details removed after review? (i.e. not wanting to appear to be giving factually incorrect information and so removing what was not on the BWC footage). Alternatively, we may ask whether participants who were less confident after their initial statement were more likely to change their statement in light of the footage?

To unpack these questions, we performed post-hoc analyses looking at whether low confidence following the initial statement predicted statement inconsistency / number of details changed in the final statement (in response to concern [iv]). This analysis could only include those in the initial statement condition who reviewed their footage (*N* = 24). Additionally, we looked at whether confidence following the initial statement predicted the number of off-camera details included within the final statement, the number of correct details removed or the change/decline in off-camera details between initial and final statements (in response to concern [v]). Here, it may be expected that participants who were less confident with their initial statement altered their final statement to more closely align with their BWC footage (i.e. making more changes, or removing off-camera details). No significant findings emerged in regression analyses asking any of these questions (*p*s > .423). This suggests that confidence is not a predictor of participants making changes to their initial statements, at least not within our sample. This study has discovered what we term a camera conformity effect, in which a statement of memory for an event mirrors recorded footage, but we invite future research to address reasons why this may occur, whether footage impacts memory or just an individual’s decision in what to include in a report.

In sum, it is difficult to distinguish whether participants removed off-camera details because they did not wish to contradict their footage, or because review had actually altered their memory for the event. Within the manuscript, we have included phrasing throughout that fits with both interpretations by emphasising footage can impact memory or what to report in a statement of memory (including page 1: “may affect a police officer’s statement about an event”, page 21: “*accounts* of memory”, page 23: “video may shape the way we come to remember *or* report an event” and page 24: “footage may shape what they *choose* to recall”).

To address this further, we have acknowledged this alternative explanation directly in the discussion. For example, we refer back to our addition on page 24, and on page 25, we have included: “ Whether body-worn video shapes episodic memory or the wearer is motivated to achieve camera consistency, this adds a layer of difficulty in understanding an officer’s in-the-moment perceptual experiences in order to rationalise their own actions (as per the direction within Graham vs. Connor, 1989).”

***Additional comments/suggestions***

***P.4, lines 79-81. Please expand and explain this statement. It is referenced, but I think an additional couple of sentences explaining to readers what the ‘problem’ is would improve this paragraph. In fact, this entire paragraph needs to be unpacked because this paragraph is presenting the rational for this research. The final sentence requires explaining at the very least.***

Further details have been included within this paragraph on page 3 to explain lines 79-81 (now lines 65-69) and the final sentence (now lines 70 - 72). Hopefully this highlights the issue with greater specificity, namely, that psychological research on body-worn cameras is minimal and as it relates to report-writing/evidence collection and memory for events, absent from the literature. In turn, this has resulted in little-to-no guidelines or empirical evidence for police forces to understand what works when incorporating body-worn video into statement-writing.

The manuscript now includes: “Consequently, there are currently no evidence-based guidelines to inform police forces on best practice of body-worn video use within report-writing [7, 16]. In light of several forces mandating their officers record all citizen encounters, body-worn video can quickly prove costly in terms of data storage and hours officers must spend reviewing incidents [15]. Therefore, whilst body-worn cameras may be an effective tool to enhance policing, how these tools are implemented may influence their benefits and thus warrants investigation. An exploration of the different reviewing and reporting practices is one area that would facilitate incident-reporting.”

***Line 105 - ‘Memories of these details are then subject to further impairment following a period of delay’ the word ‘are’ should be replaced with ‘may’, because this is not always the case.***

This suggestion for the change of wording (‘are’ to ‘may’) has been included within the manuscript.

***Line 120 - insert ‘mentally’ return, otherwise this suggests physically return, which is not what is meant here.***

The adjective ‘mentally’, as suggested, has been included on page 4 to enhance the clarity of writing.

***Paragraph commencing line 120 needs to be strengthened. The theoretical rational is weak, and should, in my opinion, be better explained and unpacked. At present the theory underpinning the rationale for this research is woolly, and rather tenuous. Suggest looking at the literature on the importance of cues for episodic memory, also. Please add some empirical research citations in this paragraph, rather than relying on books/books chapters, which are quite dated. Spreading activation needs to be explained and fully referenced: the challenges as well as the potential support for your rationale.***

Further theoretical details, supported by empirical research, have been included on page 5. The suggested paragraph has been converted into two: one to outline how photographs serve as retrieval cues for episodic memory and the second to unpack the spreading activation theory of memory in greater depth and how it may tie in with the current study and especially the reporting of off-camera details.

The two paragraphs on page 5 now read as follows:

“Photographs have proven powerful retrieval cues for episodic memory within individuals with memory impairments and cognitively healthy individuals (see [35] for a review). Viewing a photograph can strengthen a memory trace for an event via retrieval practice or rehearsal prior to an individual being tested [36–38]. Moreover, a photograph’s rich perceptual sensitivity and personal salience may facilitate the reconstruction or reactivation of a more coherent representation [39, 40]. In line with this, individuals have frequently reported that reviewing photographs have evoked feelings of ‘reliving’ that event [41–45], rather than merely ‘knowing’ something has occurred because the photograph says so [41, 46]. Similarly, body-worn video enables an officer to mentally return to the scene of an incident, reinstating the context in which they originally encoded various memories for that event (encoding specificity principle; see [47]). As body-worn video is self-generated [48] and shares a significant overlap with the original encoded memories of the officer recording the event [49], it is likely to be a highly effective retrieval cue for episodic memory.

Body-worn video may not only strengthen the activation of details within memory, but also details these are bound to or associated with beyond what the camera has recorded. For instance, spreading activation theory describes memory as being organised in a distributed and associative network [50, 51]. Therefore, a visual detail viewed onscreen will be activated in memory where it has been encoded within its spatial context (location, what was nearby), temporal context (timing within a sequence of events, what occurred before and after), social context (who was present), cognitive context (thoughts, motivation for actions) and emotional context [39]. The co-activation of different components within the event increases the likelihood of further components being activated and bound together to form a single coherent episodic memory [52, 53]. Without review, these additional details may have been inaccessible or below a threshold required to be retrieved from memory [33]. This suggests that reviewing body-worn video may elicit a more complete episodic memory trace for an experienced event, facilitating retrieval of details on and off-screen.”

***The gist versus verbatim literature is also relevant to this paper.***

Due to the length of the paper, we have opted to not discuss fuzzy-trace theory (Brainerd & Reyna, 2005; Reyna & Brainerd, 2011) in depth. We agree that body-worn video may help to strengthen verbatim traces that have decayed within memory by providing visual cues. However, we believe this would be helpful in forming hypotheses relating to whether participants included information they did not see / ‘misinformation’ as you have suggested, as presumably, details that an officer saw on their footage that they did not explicitly remember would likely be highly consistent with their gist trace for that event and thus likely to be incorporated into memory. We also believe inclusion of source misattribution would be insightful here, however, this paper does not investigate these questions.

***Does body worn camera footage ‘rewrite’ and officer’s memory? Or does it lead to the officer altering their statement because they begin to doubt their own memory, and loose confidence in their cognition. This is also a question that needs to be considered/highlighted - see lines 177-179.***

We hope we have provided greater clarity to this point under concerns [iv] and [v].
